# Differential signal sensitivities can contribute to the stability of multispecies bacterial communities

**DOI:** 10.1186/s13062-017-0192-3

**Published:** 2017-09-15

**Authors:** János Juhász, Dóra Bihary, Attila Jády, Sándor Pongor, Balázs Ligeti

**Affiliations:** 1 Faculty of Information Technology and Bionics, Pázmány Péter Catholic University, Práter Street 50/A, Budapest, H-1085 Hungary; 2 Present address: RC Cancer Unit, Hutchison/MRC Research Centre, University of Cambridge, Hills Road, Cambridge, CB2 0XZ UK; 3 Institute of Medical Microbiology, Semmelweis University, Nagyvárad square 4, Budapest, H-1089 Hungary

**Keywords:** Microbiome, Quorum sensing, Swarming, Self-restraint, Response threshold, Antibiotic production, Agent-based modelling

## Abstract

**Background:**

Bacterial species present in multispecies microbial communities often react to the same chemical signal but at vastly different concentrations. The existence of different response thresholds with respect to the same signal molecule has been well documented in quorum sensing which is one of the best studied inter-cellular signalling mechanisms in bacteria. The biological significance of this phenomenon is still poorly understood, and cannot be easily studied in nature or in laboratory models. The aim of this study is to establish the role of differential signal response thresholds in stabilizing microbial communities.

**Results:**

We tested binary competition scenarios using an agent-based model in which competing bacteria had different response levels with respect to signals, cooperation factors or both, respectively. While in previous scenarios fitter species outcompete slower growing competitors, we found that stable equilibria could form if the fitter species responded to a higher chemical concentration level than the slower growing competitor. We also found that species secreting antibiotic could form a stable community with other competing species if antibiotic production started at higher response thresholds.

**Conclusions:**

Microbial communities in nature rely on the stable coexistence of species that necessarily differ in their fitness. We found that differential response thresholds provide a simple and elegant way for keeping slower growing species within the community. High response thresholds can be considered as self-restraint of the fitter species that allows metabolically useful but slower growing species to remain within a community, and thereby the metabolic repertoire of the community will be maintained.

**Reviewers:**

This article was reviewed by Michael Gromiha, Sebastian Maurer-Stroh, István Simon and L. Aravind.

**Electronic supplementary material:**

The online version of this article (doi:10.1186/s13062-017-0192-3) contains supplementary material, which is available to authorized users.

## Background

Bacteria are the most widespread life forms on Earth that populate every habitat, including the surfaces of the human body. [1–3]. In most cases bacterial cells live in large multispecies communities in which they compete for nutrients and space, but at the same time they also cooperate with each other via chemical materials secreted into the environment. One well-studied mechanism whereby bacterial cells can communicate and cooperate with each other is quorum sensing (QS) [4, 5]. QS is based on the ability of cells to respond to a chemical signal that they themselves release into the environment. As the environmental concentration of the signal will be higher if many similar cells are present, this simple mechanism allows cells to indirectly sense population density. In this manner a population can turn on and off metabolic functions in a synchronized manner, which enables it to solve problems that individual cells cannot tackle, such as colonizing habitats, infecting host organisms etc.

One of the simplest QS systems is the N-acyl homoserine lactone (AHL) based communication present in Gram negative bacteria [6]. In a typical case, such as present in *Pseudomonas aeruginosa*, cells constantly produce a low level of AHL [7, 8]. Just to take a simple hypothetical example, if the extracellular AHL concentration exceeds a threshold level, cells turn on the metabolically expensive production of an enzyme. When the enzyme concentration reaches a critical level, it will digest protein nutrients present in the environment, and the liberated amino acids will allow cells to upgrade their metabolic activities. In reality, there are over 70 AHL molecular signals known in various Gram negative bacteria [9], and a typical bacterium has several QS systems [10–12], which makes the experimental study of QS quite difficult. Also, not only enzyme production but a large variety of metabolic or regulatory functions can be activated by QS [5, 11], so general conclusions cannot be easily reached from the study of individual species.

It is a well-known property of AHL signalling that bacterial species often react to various chemical signals emitted by other bacterial species [13]. From the protein structural point of view this is not a surprise, since the AHL binding pocket of an enzyme that can bind a particular AHL molecule may also bind a related AHL molecule, even though in a less efficient manner [14]. As a result, a bacterium, harbouring a certain AHL receptor molecule will be able to respond to a variety of AHL signals of related structures. The reason and the significance of this quite widespread phenomenon are currently unknown. The main goal of this work is to clarify whether or not differential sensitivity to various signals can contribute to the maintenance of a bacterial community.

Finally, a natural but rarely asked question regarding microbial communities refers to the coexistence of species differing in their fitness. Namely, a typical interspecies microbial community in nature harbours over ten thousand bacterium species that must necessarily differ in their growth rates. Still, the fittest species does not simply outcompete the slower growing ones, but a certain number of slow-growing species are continuously present over long periods of time [15]. One can consider this phenomenon as the self-restraint or moderation of the fitter species since slower growing species will not be eliminated as one could expect from the classical competitive exclusion principle of Gause [16]. Previously we have shown that sharing of signals and nutrients can in fact contribute to the stable cooperation of different species [17] and that complex model communities can even exhibit territorial defence [18]. Here we ask the specific question whether or not the differential response to the same chemical signal can be the molecular basis of such seemingly complex concepts as self-restraint or moderation of bacterial species.

In this work we used agent-based simulations to study the role of multiple signal sensitivities in bacterial communities. We found that differential response thresholds provide a simple and elegant way for keeping slower growing species within a community. Also, they can lead to stable coexistence between antibiotics-producing and antibiotics-sensitive cells. We hope this research can help one to better understand the dynamics of complex microbial communities.

## Results and discussion

### Modelling framework and competition outcomes

We set up competition experiments by placing randomly an equal number of agents representing the two competing species at the beginning of a longitudinal 2D surface “track” covered by the nutrient (see Methods). At the beginning, all cell agents were in the solitary (ground) state. When the simulation started, the cells started feeding, moving randomly, dividing and producing a low amount of diffusible communication signal (S) at a rate corresponding to the solitary state. As the concentration of the signal in the environment reached a certain threshold, the corresponding cells switched to an active state, and started to produce a public good, which we term a cooperation factor (F) (in the example cited in the introduction this was an enzyme). When the factor in the environment reached a threshold concentration, the cells switched to the swarming state i.e. they increased food intake, movement, as well as the production of signal and cooperation factor. As a result, agent communities have been formed that proceeded along the track. The region where signal and factor concentrations are high and most cells situated is called the active zone [19]. Agent communities were considered stable if their population size was practically constant for at least 20 generations. The constant population size indicated that the nutrient capacity of the environment was reached. In other cases the communities were not able to form viable microbiome, they either remained at the starting point or they collapsed after a short growth period. In previous studies we observed a few further competition phenotypes including homogeneous and spatially separated mosaic-like colonies [17].

**Fig. 1:**
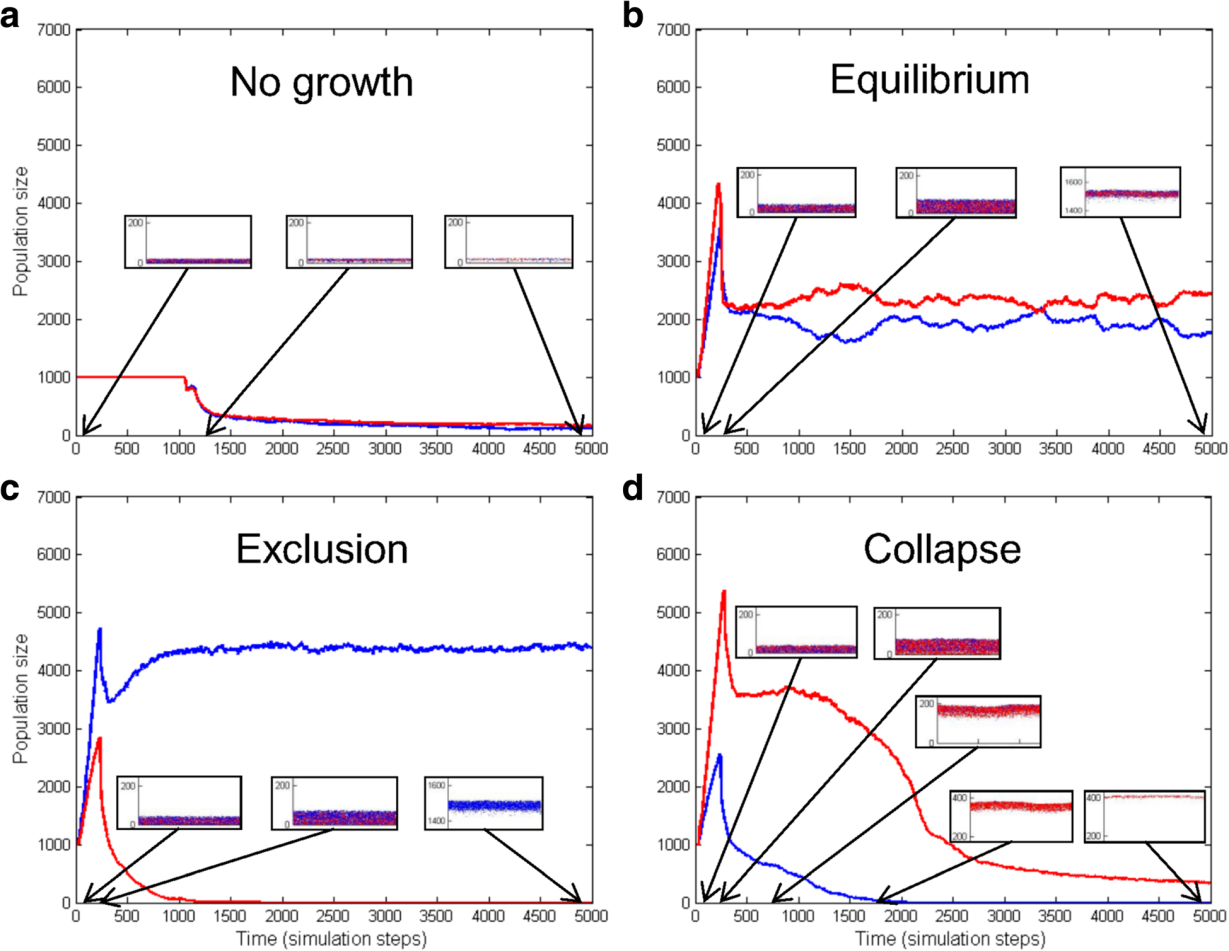
Competition phenotypes of two species simulations. **a** There is no growth if species are unable to grow under the given conditions, e.g. they are incapable of quorum sensing; **b** stable coexistence of two different cell types; **c** exclusion of a less fit species by a fitter one; **d** collapse of a community. *Blue lines* indicate the cell numbers of the first (in case of **b**, **c**, **d** WT) species and the *red lines* the cell number of the other species as a function of time (in simulation steps). The small inserts show the population on the two dimensional longitudinal track in the time points indicated by the arrows. *Blue* and *red dots* represent the positions of agents from different species

With the particular agent pairs used in this study, we observed only a few competition phenotypes, schematically shown in Fig. 1. Figure 1a shows a situation when a population cannot grow under the given conditions. In this case cells remain in their solitary (ground) state, consume the available nutrient and then the populations starve out when the nutrient pool is depleted. Figure 1b demonstrates a scenario where two species can stably coexist. In this case, the cells form a homogeneous community. Figure 1c shows a situation where a fitter species with faster division outcompetes another, slower growing one. This is a typical case of competitive exclusion [16]. The results are the same if the fitter species spends less energy (from nutrient uptake) for communication and cooperation with signal and factor compounds. Finally, Fig. 1d shows a scenario in which the fitter species (red) is not viable in itself. This is typical in the case of cheater phenotypes that can survive only using the signals and cooperation factors produced by another, slower growing species. In this case the population collapses after a short growth phase.

### Higher response threshold induces self-restraint

Let us imagine the competition of two species, species 1 and 2 that have identical parameters, for instance they react to the signal at a threshold concentration T_1_. This means that at this threshold value the cells switch to faster metabolism and faster growth. As all parameters are equal, both populations grow at an equal rate. Now let’s raise the response threshold of species 2 to a higher value T_2_. At the beginning of the simulation, the signal concentration in the environment is zero. As the signal concentration starts to grow, it will first reach T_1_, so species 1 will switch from a dormant, ground state to a more active, faster growing state, while species 2 will continue to remain in the ground state. As the signal concentration will reach T_2_, species 2 will also switch to the active state, but between T_1_ and T_2_ concentration species 2 gives an advantage to species 1.

**Fig. 2:**
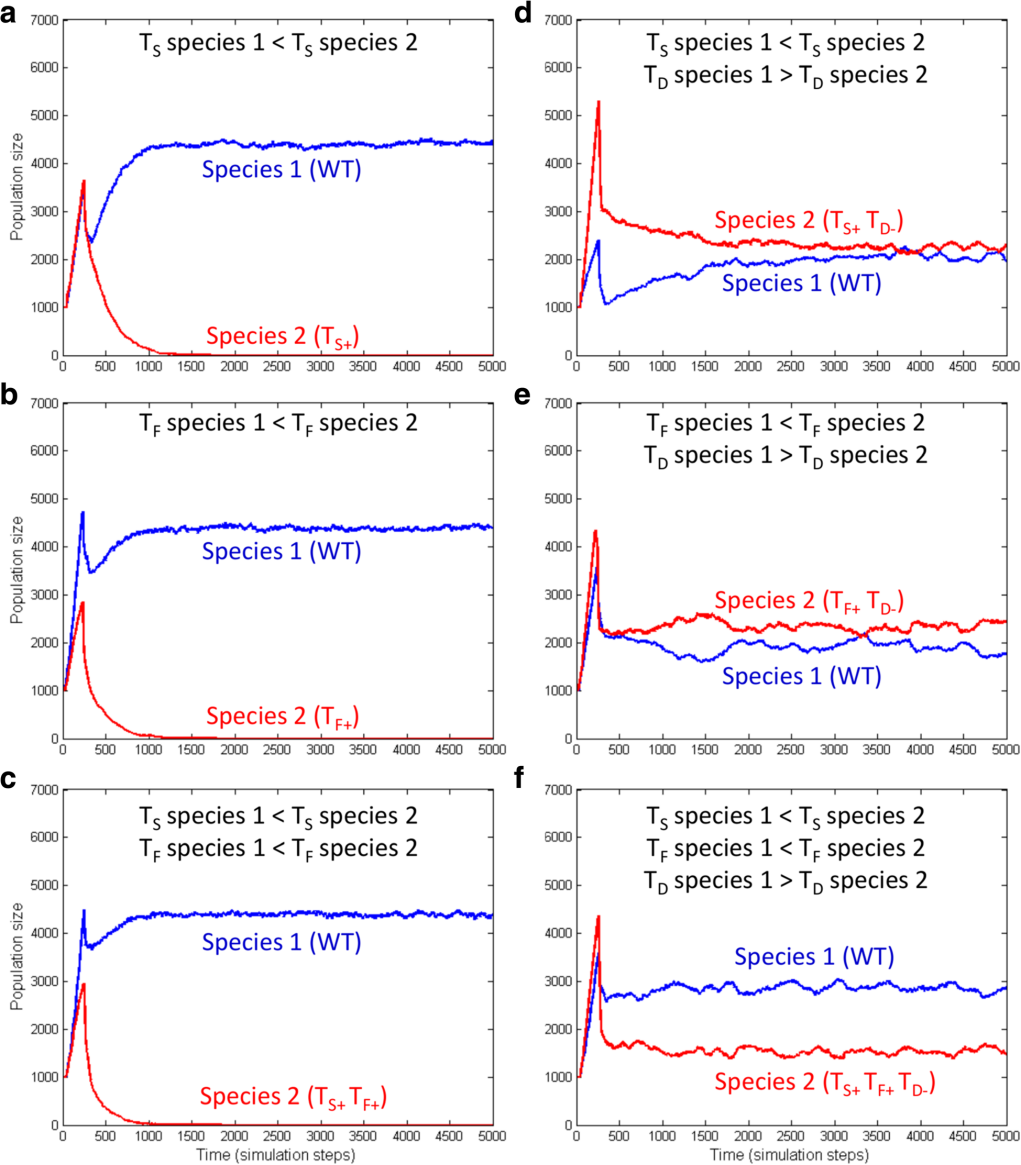
Competition between WT species 1 (*blue*) and species 2 with modified threshold values (*red*). **a** WT species 1 and higher signal threshold (T_S+_) species 2, leads to exclusion of species 2; **b** WT species 1 and higher factor threshold (T_F+_) species 2, leads to exclusion of species 2; **c** WT species 1 and higher signal and factor threshold (T_S+_ T_F+_) species 2, leads to exclusion of species 2; **d** WT species 1 and higher signal threshold and higher fitness (T_S+_ T_D-_) species 2, leads to coexistence of species 1 and species 2; **e** WT species 1 and higher factor threshold and higher fitness (T_F+_ T_D-_) species 2, leads to coexistence of species 1 and species 2; **f** WT species 1 and higher signal and factor threshold and higher fitness (T_S+_ T_F+_ T_D-_) species 2, leads to coexistence of species 1 and species 2

The previous considerations indicate that higher response thresholds lead to decreased fitness in a certain signal concentration range, namely between T_1_ and T_2_. Increasing the communication signal threshold, the cooperation factor threshold or both, the affected species decreases its fitness, which could lead to its exclusion. This is exactly what we saw in our simulation experiments. We increased the signal threshold (Fig. 2a), the factor threshold (Fig. 2b) or both (Fig. 2c), respectively, for species 2. The populations with increased signal and factor thresholds were viable when grown alone. In co-cultures with wild type (WT) species 1, the latter was a clear winner, which indicates that higher response threshold is a disadvantage.

### Differences in response thresholds can lead to stable cooperation

As a next step let’s design a competition experiment in which two competing species differ in their growth rates. This is a natural scenario since it is difficult to imagine that two species grow exactly at the same pace (note that under the studied conditions, populations of dormant cells do not grow). Continuing the thought experiment in the previous section, first suppose that species 2 has a lower growth rate. Since this species has a higher signal response threshold, it is already at a disadvantage. Diminishing its growth rate will further increase this disadvantage so the picture will not change dramatically. Now suppose that species 2 has a higher growth rate. In this case, species 2 will be at a disadvantage below its response threshold T_2_, but will be at an advantage above the threshold level (more exactly, the two species will be inactive below T_1_, species 1 will grow alone between T_1_ and T_2_, and both species will grow above T_2_ but species 2 will grow faster). This situation corresponds to one of the classical definitions of population equilibria, since on one side of the threshold T_2_, species 2 will grow faster while on the other side, species 1 will be the fitter one. This leads to a fluctuation around an equilibrium population ratio. We used the WT species 1 and species 2 agents from the previous experiment to test this hypothesis. The growth rate of species 2 was increased by decreasing its division threshold (see Methods). While all of these species could swarm alone, the results showed that they were capable of stable coexistence when grown together (Fig. 2d–f). In other words, the sole difference between the right and left panels is that in the right panels, species 2 divides faster – i.e. is more fit – than species 1. This fitness difference seems sufficient to induce a stable coexistence between the two species. With respect to the range between T_1_ and T_2_ we note, that in our simulations, slight differences were sufficient to create an equilibrium while in nature the differences can be substantially higher, sometimes more than an order of magnitude. We think that phenomenon may be a factor underlying natural population equilibria in bacterial communities.

### Differential signal thresholds can stabilize equilibria even between antibiotics producing and sensitive strains

**Fig. 3:**
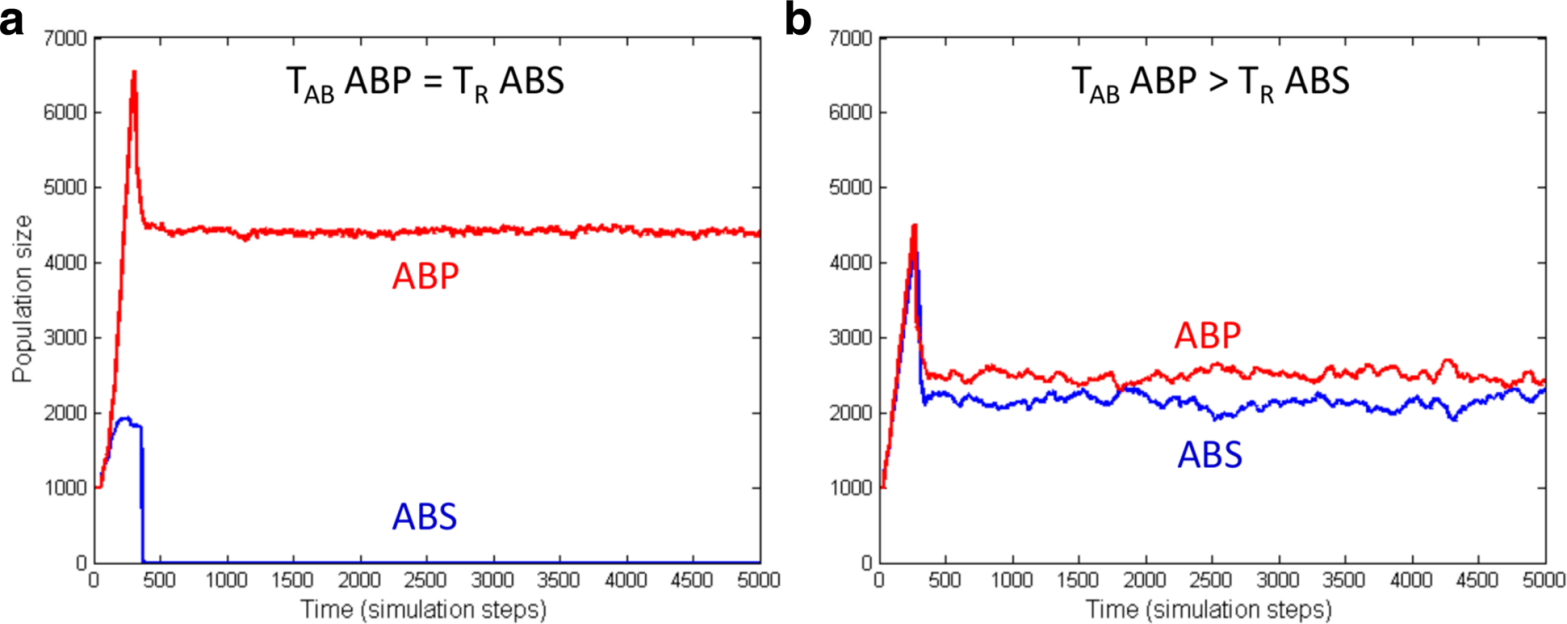
Competition between AB sensitive (ABS)(*blue*) and AB producing (ABP) (*red*) populations. Raising the response threshold (T_AB_) of an antibiotic producing eavesdropping species leads to stable coexistence with the target ABS species (the members of which are activated at threshold T_R_). **a** eavesdropping with equal signal thresholds, leads to exclusion of the eavesdropped species; **b** eavesdropping after raising the threshold of the eavesdropper for foreign signal leads to coexistence between the species

“Chemical warfare” is often part of bacterial competition scenarios. For instance, the species *Chromobacterium violaceum (Cv)* emits an antimicrobial (AB) upon sensing the QS signal of *Burkholderia thailandiensis (Bt),* which prevents the invading *Bt* from penetrating the habitat of *Cv* [20]. In other terms *Cv* eavesdrops on the signal of *Bt* and starts a chemical attack as soon as the signal of *Bt* reaches a threshold concentration. Previously we showed that eavesdropping provides a unilateral advantage for the eavesdropping species [17]. As signal-activated chemical attacks are widespread in the bacterial world [21, 22], the question emerges if coexistence can still be achieved by modulating the response threshold of the eavesdropping species. To answer this question, we set up a competition experiment wherein the AB sensitive (ABS) species was slightly fitter than the AB producing (ABP) species. In this case, the AB producing cells were the clear winners (Fig. 3a). However, raising the response threshold of ABP against ABS imposed self-restraint on the AB producer and a typical fluctuating equilibrium emerged (Fig. 3b). The reason is that below the response threshold, AB sensitive cells were fitter than AB producers, while above the response threshold the situation reversed.

## Conclusions

Bacteria living in the same niche often respond to identical chemical signals [23] but have vastly different response thresholds [15]. It is presently unknown why such properties should be maintained during evolution. Here we carried out computer simulations showed that different response thresholds can lead to stable population equilibria between competing species. The clue in our case was that the fitter species can demean self-restraint by switching to its metabolically active state at a signal concentration which is higher than the response threshold of the competing species. In such a way, the less fit competing species will have an advantage in a given signal or cooperation factor concentration range, while the fitter species will have growth advantage above that concentration range. This will give rise to population sizes fluctuating around an equilibrium level.

Natural microbial communities rely on the stable coexistence of different microbial species with different fitness values. Less fit, i.e. slower growing species can be crucial for the entire community if they produce metabolites essential for others. Keeping such less fit species is in the interest of the entire community. A simple and plausible method for preserving them is the use of different quorum sensing response thresholds. Higher response thresholds can be considered as a self-restraint mechanism of fitter species that helps maintaining the metabolic and functional repertoire of a microbiome via allowing useful but less fit members to grow under certain circumstances.

We suppose that the origin of different response thresholds is evolutionary, for instance a result of mutations within the binding sites of quorum sensing receptors. Cells that are capable of working together, could recruit each other in the process of community formation [15], or exclude other “incompatible” species from the community. Another important benefit of applying different signal and factor thresholds within a community, besides the division of work, is the faster and smoother adaptation to changing environments. If some parameters of the environment change, the fitness of the community, optimised to these conditions, decreases. If the community contains identical cells, adaptation to the new conditions must be an evolutionary process, based on mutations and natural selection. But if the community contains different cell populations, it can adapt through changing the population ratios which is a much faster process that takes place on an ecological rather than evolutionary time-scale.

## Methods

### Modelling framework

For studying the effects of different response thresholds, we employed an agent-based model of quorum sensing (QS) previously developed in our group [17, 19, 24], implemented in MATLAB programming language and using the basic parameters summarized in Additional file 1: Tables S1-S8. Briefly, this model represents bacterial cells as computational agents that randomly move on a 2D surface. The agents consume nutrients, and invest the energy gained in this way into producing signal molecules (S) as well as cooperation factors (F). Nutrients, signals, cooperation factors are diffusible materials that freely diffuse on the 2D plane. At the beginning of the simulation the cells are placed on one end of a 2 dimensional half-closed longitudinal track that is open at the opposite end and has periodic boundary condition on its two sides. This modelling setup corresponds to the growth of a single dendrite of a bacterial colony placed on an agar plate. The simulations were carried out with our agent based model described in [17], parameters summarized in Additional file 1: Tables S2-S3. In a typical simulation run, 1000 cells of each of the two competing species were randomly placed to the beginning of the longitudinal track, and the simulation was left to proceed for 5000 steps (approximately 110 generations).

### Models with elevated response thresholds

The key elements in our simulations are the two response thresholds T_S_ (signal threshold) and T_F_ (factor threshold) at which the models switch to another state. We designed models with elevated response thresholds (Table 1). The elevated thresholds were selected empirically in such a way that the difference between populations should appear within 5000 simulation steps, i.e. the standard length of our simulation experiments. The elevated values are 2–4 folds higher than the basic levels, in nature we find much bigger differences [15]. The fitness in our models is determined by the division threshold, T_D_, which corresponds with the energy content of the cells that allows them to divide. The models with elevated fitness had a lower division threshold that allowed them to divide faster.

**Table 1:** Modified growth rate, signal and factor threshold parameters of the self-restraint experiments

Name of the model	Division threshold (T_D_)	Signal threshold (T_S_)	Factor threshold (T_F_)
Basic model, WT, (blue in Fig. 1b,c,d, Fig. 2)			
WT	12	10	10
Models with elevated response thresholds (red in Fig. 2a,b,c)			
T_F+_	12	45	10
T_S+_ species 2	12	10	20
T_F+_T_S+_ species 2	12	45	20
Models with elevated response thresholds and increased fitness (red in Fig. 2d,e,f)			
T_F+_T_D-_	6	45	10
T_S+_T_D-_	6	10	20
T_F+_T_S+_T_D-_ species 2	6	45	20

### Antibiotics production

Antibiotic (AB) production was introduced into the models by designing antibiotic producing (ABP) and antibiotic sensitive (ABS) agent types capable of QS. ABS agents are wild type-like models that are sensitive to a diffusible antibiotic, AB. When the concentration of AB exceeds a threshold, ABS cells fall back to the ground state i.e. they will not be viable under the given modelling conditions. In other words, ABP cells eavesdrop on ABS cells and attack them if the local density of ABS cells is above a certain level. The critical parameters in these simulations were the threshold value T_R_, at which ABS cells switched to a higher metabolic state, and T_AB_ at which ABP cells started to produce antibiotics (Table 2). We tested two kinds of scenarios. In the first one (Fig. 3a), “T_AB_ = T_R_”, AB production started at the same signal concentration at which ABS cells switched to a higher metabolic state. In the second one (Fig. 3b), “T_AB_ > T_R_”, antibiotic production started at a higher value, allowing ABS agents to grow before AB production would have started. Note that in this system, AB production can be meaningfully tested only if the ABS cells are fitter than ABP cells. Otherwise ABS cells are excluded even without AB action. ABS models were made fitter by assigning a division threshold lower than that of ABP cells (i.e. 11 instead of 12).

**Table 2:** Modified growth rate and antibiotics production threshold parameters of the antibiotics production experiments

Name of the model	Division threshold	AB production threshold (T_AB_)	Response threshold (T_R_)
Model with antibiotics sensitivity (blue in Fig. 3)			
ABS	11	-	10
Model with antibiotics production T_AB_ = T_R_ (red in Fig. 3a)			
ABP	12	10	-
Model with antibiotics production T_AB_ > T_R_ (red in Fig. 3b)			
ABP	12	30	-

## Reviewers’ comments

### Reviewers’ report 1: Michael Gromiha, Indian Institute of Technology Madras, India

In this work, the authors addressed a fundamental question of bacterial communities. The stability of microbial communities is one of the difficult questions of biology today. Namely, communities found in various habitats are varied, so it is not only notoriously difficult to study them by experimental methods, but also it is not easy to pinpoint principles that are applicable to different communities. Juhász et al. chose a phenomenon known in quorum sensing bacteria: different species react to the signal of each other but at vastly different response thresholds. The authors showed using generic agent-based models that the existence of differential signal response thresholds can contribute to the stability of bacterial communities since population equilibria may exist in a large part of the parameter space. The work is interesting. The manuscript is well written and the figures are adequate.

Minor: The phrase “As a thought experiment” could be deleted or rephrased.

Author’s response: *Thank you for the advice, the mentioned part was rephrased.*

### Reviewers’ report 2: Sebastian Maurer-Stroh, Bioinformatics Institute (BII), a*STAR, Singapore

Co-existence of species in bacterial communities is an interesting but complex question that can be addressed in a variety of models. The manuscript “Differential signal sensitivities can contribute to the stability of multispecies bacterial communities” by Juhasz et al. describes agent-based simulations to study stability of bacterial communities competing for the same resources in a 2D periodic boundary setup. Their model includes parameters for quorum sensing with signalling molecules and cooperation factors with a signal threshold T(s) and factor threshold T(f) controlling when agents switch between solitary, active and swarming states and show that the system behaves as expected in standard scenario. Specifically, they try to answer the question how 2 species with differing division fitness could co-exist when the competitive exclusion principle would simply suggest that the fitter species survives and the less fit would die out. They propose and show that differences in signal concentration thresholds would be a possible solution to allow for co-existence of these species. This works because the fitter species switches of state later which gives an advantage to the less fit species at low signal concentrations. If the parameters are chosen carefully, this can result in a homeostasis between the species. The authors note that this would correspond to self-restraint by the fitter species. They show a similar outcome of a stable community for antibiotic producing vs. sensitive species if the antibiotic is only produced above a higher response threshold. The flow and message of the manuscript is clear. I only have minor comments:

1. Small inserts in Figure 1, please explain what they should show.
2. Page 4: delete “of” in “classical of competitive”.

Author’s response: *Thank you for the suggestions, the mentioned parts were explained and corrected in the text of the article*.

### Reviewers’ report 3: István Simon, Institute of Enzymology, Hungary

The ms. of János Juhász et al. (“Differential signal sensitivities can contribute to the stability of multispecies bacterial communities”) deals with a fundamentally important question, the stability of multispecies microbial communities. This phenomenon is hard to study by experimental methods since communities vastly differ in terms of species composition and population ratios so the conclusions can not be easily generalized to other communities. The authors thus chose computer modelling, notably agent based models. This ms deals with a specific question related how signal molecules can contribute to the stability of a community. It is known in the field of quorum sensing, that species present in the same environment often react to the same signal molecule but at vastly different response thresholds. The authors show that as a result, stable communities can form in a sufficiently large part of the parameter space. I think this is a nice and simple conclusion. I find it particularly interesting that seemingly complex anthropomorphic concepts such as moderation or self-restraint can be traced back to simple physico-chemical and regulatory notions such as response thresholds.

The authors should nevertheless make clear throughout the manuscript that “moderation” and “self-restraint” are phenomenological conclusions, i.e. the result, not the cause.

Author’s response: *Thank you for mentioning this point. It is important to note that in this paper, we would like to highlight some potential mechanisms that could explain the stable coexistence of different competing species in complex microbial communities. So we state that the differences in quorum sensing response thresholds could be a cause of this phenomenon. “Moderation” and “self-restraint” are anthropomorphic terms – here we show that these behaviours can result from simple physicochemical, regulatory principles. We now have made this clear in the manuscript.*

Also, I think the authors should add a paragraph related to their original question, why differential response thresholds are present in nature. In my view, these can be fixed by evolution, or rather formed by recruitment on the spot, i.e. species having these characteristics will preferentially recruit each other.

Also, the authors may want to add that differential signalling thresholds allow the community to respond by modifying their population ratios (ecological time-scale), which is more efficient and swift than simple collapse whereby the community is simple “selected out” (evolutionary time-scale).

Author’s response: *Thank you for the important considerations and thoughts about the origin and benefits of diverse response thresholds, we now mention and discuss them at the conclusion part of the manuscript.*

### Reviewers’ report 4: L. Aravind, NCBI, USA

The manuscript “Differential signal sensitivities can contribute to the stability of multispecies bacterial communities” submitted by Juhász et al. addresses the question whether or not the differential response characteristics of bacterial species to the same quorum sensing signal can influence the coexistence of bacterial species. Given the importance of intra-specific biological conflicts in establishment of microbial communities obtaining constraints for this via theoretical models would be particularly useful. Bacterial species that respond to an environmental signal at a lower concentration threshold can in principle easily outcompete others. Hence, some of the other species may get lost from the community. If such a species happens to carry a metabolic function crucial to the community, the survival of the entire community will be jeopardized. However if a fitter species responds at a higher signal concentration level, the less fit species will still survive i.e. the metabolic repertoire of the community will be maintained.

The manuscript is clearly written but the authors may want to discuss the below points:

1) The authors propose that competitive exclusion of a less fit species that would be eliminated as per the Gause principle is precluded by the higher threshold of signal sensing. As the species shuttle between fitter and less fit regimes, it may be interesting to compare their average fitness calculated over entire the simulation period so as to show if and how the Gause principle is violated.

Author’s response: *The Gause principle is only briefly mentioned in the paper and without explicitly mentioning the fact of violation. Namely, in our opinion, the principle is not violated in this system, even though the average fitness values can be different. What we see (data not shown, see also *[17, 19, 24]*)* *is spatial separation, the species with faster division (fitter) occupy most of the swarming zone, and gradually exclude the slower growing (less fit) species. Nevertheless at the front region of the swarming zone the slower growing cells have advantage, because the other species cannot swarm there due to its higher quorum sensing thresholds. In other terms, spatial heterogeneity is observed, with the less fit species being excluded from only a given spatial region. Spatial heterogeneity had been invoked as an explanation why less fit species can avoid extinction (see* e.g. [25, 26]*) – this seems to be the case even in our highly simplified model system. These indicates that spatial exclusion could be crucial for understanding the Gause principle and the behaviour of apparently excluded species surviving in remote niches, and it can be observed even in simple, agent-based systems*.

2) Moreover, the Gause principle was originally formulated in an intraspecific context but the authors are using models such as Chromobacter and Burkholderia which inter-specific competition. They would want discuss the generalization of the Gause principle for such scenarios.

Author’s response: *Our models are course approximations and at this level of abstraction, intra and interspecific contexts do not separate sharply. The existence of spatial heterogeneity and the swarming behaviour of the colonies also differentiate our simulation setup from the environment where Gause principle was originally applied*.

3) The authors propose this “restraint” mechanism as playing a role in survival of bacterial communities. What would change if this mechanism did not exist? Is it conceivable that such mechanisms are selected for in the first place only in scenarios where community collapse imposes a much greater cost than the benefit from eliminating the rival organism?

Author’s response: *Yes, this is true and an explanation is now added to the text. We note that the described “restraint” mechanism is crucial in our (quorum sensing driven swarming) system for the formation of spatial heterogeneity and permanent coexistence of different species. Nevertheless community collapse is the worst scenario here, because it leads to a dramatic decrease in population size and an end of swarming, so it has much greater cost than swarming in the company of a rival organism*.

It should be Gause principle? That is how the author of the original paper is spelt in English but it was Gauze in Russian?

Author’s response: *Yes, this is true. We now use the English spelling which is more common in the scientific literature.*

*Finally we thank all four reviewers for their work and useful, thought-provoking comments.*

## Additional file

Additional file 1: Basic parameters of the simulations. (PDF 271 kb)

## Abbreviations

AB: Antibiotics
ABP: Antibiotic producing
ABS: Antibiotic sensitive
AHL: N-acyl homoserine lactone
Bt: *Burkholderia thailandiensis*
Cy: *Chromobacterium violaceum*
F: Factor
QS: Quorum sensing
S,S1,S2: Signal
T1,T2: Response threshold
T_AB_: Antibiotic production threshold
T_D_: Division threshold
T_F_: Factor threshold
T_R_: Response threshold
T_S_: Signal threshold
WT: Wild type
