# Supplementary material for: Differential signal sensitivities can contribute to the stability of multispecies bacterial communities
Source: Biol Direct. 2017 Sep 15;12:22. doi: 10.1186/s13062-017-0192-3 (PMC5602943; doi:10.1186/s13062-017-0192-3)
Supplement: Additional file 1: — Basic parameters of the simulations. (PDF 271 kb) [file 13062_2017_192_MOESM1_ESM.pdf]

Additional file

Basic parameters of the simulations

Table S1: Configuration file of the quorum sensing model–main program parameters

Simulation parameters:						
Maximum number of simulation steps	5000					
Snapshot time	1					
Graphical output	yes					
Medium parameters:						
Max number of bacterium in cells	10					
Medium size (x)	250					
Medium size (y)	2000					
Lattice size (x)	50					
Lattice size (y)	400					
Produced materials:						
Initial concentration of materials in cells	0	0	0	0	0	0
Initial food concentration in cells	500					
Population:						
Number of species	2					
Number of states	4	(8 for AB)				
Initial number of bacteria (of species)	5	5				
Energy after division	0.2					
Mutation rate	-1					
Mutant number	1					
Border advancement:						
Border advancement threshold	1					
Diffusion parameters:						
Diffusion coefficients of food	0.03					
Diffusion coefficients of materials	0.02	0.05				
Decay constants of materials	0.001	0.0001				
Time step	40					
Simulation type:	single					

Table S2: Configuration file of the quorum sensing model–threshold pattern

Produced materials (binary)						Index (decimal + 1)	State identifiers of species			
P6	P5	P4	P3	P2	P1		S1	S2	S3	S4
0	0	0	0	0	0	1	1	1	0	0
0	0	0	0	0	1	2	2	2	0	0
0	0	0	0	1	0	3	3	3	0	0
0	0	0	0	1	1	4	4	4	0	0

Threshold pattern for two species with the same quorum sensing system

Table S3: Configuration file of the quorum sensing model–WT agent parameters

Threshold concentration of materials:					
P1	P2	P3	P4	P5	P6
10	10	10000	10000	10000	10000

Division threshold	12
Metabolic energy	0.1

			Amount of produced materials						Energy spent on producing materials					
State	Speed	Food	P1	P2	P3	P4	P5	P6	P1	P2	P3	P4	P5	P6
1	1.5	0.3	0.2	0	0	0	0	0	0.01	0	0	0	0	0
2	1.5	0.3	0.34	0.07	0	0	0	0	0.017	0.105	0	0	0	0
3	1.5	0.3	0.34	0	0	0	0	0	0.017	0	0	0	0	0
4	5	0.5	0.34	0.07	0	0	0	0	0.017	0.105	0	0	0	0

Table S4: Configuration file of the quorum sensing model–baseline signal producing agent parameters

Threshold concentration of materials:					
P1	P2	P3	P4	P5	P6
10	10	10000	10000	10000	10000

Division threshold	12
Metabolic energy	0.1

			Amount of produced materials						Energy spent on producing materials					
State	Speed	Food	P1	P2	P3	P4	P5	P6	P1	P2	P3	P4	P5	P6
1	1.5	0.3	0.2	0	0	0	0	0	0.01	0	0	0	0	0
2	1.5	0.3	0.2	0	0	0	0	0	0.01	0	0	0	0	0
3	1.5	0.3	0.2	0	0	0	0	0	0.01	0	0	0	0	0
4	5	0.5	0.2	0	0	0	0	0	0.01	0	0	0	0	0

Parameters of the agents with only baseline signal production (Figure 1A, collapse).

Table S5: Configuration file of the quorum sensing model–decreased material producing agent parameters

Threshold concentration of materials:					
P1	P2	P3	P4	P5	P6
38	10	10000	10000	10000	10000

Division threshold	12
Metabolic energy	0.1

			Amount of produced materials						Energy spent on producing materials					
State	Speed	Food	P1	P2	P3	P4	P5	P6	P1	P2	P3	P4	P5	P6
1	1.5	0.3	0.2	0	0	0	0	0	0.01	0	0	0	0	0
2	1.5	0.3	0.25	0.025	0	0	0	0	0.0125	0.0375	0	0	0	0
3	1.5	0.3	0.25	0	0	0	0	0	0.0125	0	0	0	0	0
4	5	0.5	0.25	0.025	0	0	0	0	0.0125	0.0375	0	0	0	0

Parameters of the species 2 agents in Figure 1D with decreased signal and factor production

Table S6: Configuration file of the quorum sensing model–threshold pattern

Produced materials (binary)						Index (decimal + 1)	State identifiers of species			
P6	P5	P4	P3	P2	P1		S1	S2	S3	S4
0	0	0	0	0	0	1	1	1	0	0
0	0	0	0	0	1	2	0	2	0	0
0	0	0	0	1	0	3	3	3	0	0
0	0	0	0	1	1	4	0	4	0	0
0	0	0	1	0	0	5	2	5	0	0
0	0	0	1	0	1	6	0	6	0	0
0	0	0	1	1	0	7	4	7	0	0
0	0	0	1	1	1	8	0	8	0	0
0	0	1	0	0	0	9	0	0	0	0
0	0	1	0	0	1	10	0	0	0	0
0	0	1	0	1	0	11	0	0	0	0
0	0	1	0	1	1	12	0	0	0	0
0	0	1	1	0	0	13	0	0	0	0
0	0	1	1	0	1	14	0	0	0	0
0	0	1	1	1	0	15	0	0	0	0
0	0	1	1	1	1	16	0	0	0	0
0	1	0	0	0	0	17	5	0	0	0
0	1	0	0	0	1	18	0	0	0	0
0	1	0	0	1	0	19	7	0	0	0
0	1	0	0	1	1	20	0	0	0	0
0	1	0	1	0	0	21	6	0	0	0
0	1	0	1	0	1	22	0	0	0	0
0	1	0	1	1	0	23	8	0	0	0

Threshold pattern for two species with the same factor but different signals and with S2 AB production against S1 signal

Table S7: Configuration file of the quorum sensing model–AB sensitive agent parameters

Threshold concentration of materials:					
P1	P2	P3	P4	P5	P6
10000	10	10	10000	10	10000

Division threshold	11
Metabolic energy	0.1

			Amount of produced materials						Energy spent on producing materials					
State	Speed	Food	P1	P2	P3	P4	P5	P6	P1	P2	P3	P4	P5	P6
1	1.5	0.3	0	0	0.2	0	0	0	0	0	0.01	0	0	0
2	1.5	0.3	0	0.07	0.34	0	0	0	0	0.105	0.017	0	0	0
3	1.5	0.3	0	0	0.34	0	0	0	0	0	0.017	0	0	0
4	5	0.5	0	0.07	0.34	0	0	0	0	0.105	0.017	0	0	0
5	1.5	0.3	0	0	0.2	0	0	0	0	0	0.01	0	0	0
6	1.5	0.3	0	0	0.2	0	0	0	0	0	0.01	0	0	0
7	1.5	0.3	0	0	0.2	0	0	0	0	0	0.01	0	0	0
8	1.5	0.3	0	0	0.2	0	0	0	0	0	0.01	0	0	0

Table S8: Configuration file of the quorum sensing model–AB producing agent parameters

Threshold concentration of materials:					
P1	P2	P3	P4	P5	P6
10	10	30	10000	10000	10000

Division threshold	12
Metabolic energy	0.1

			Amount of produced materials						Energy spent on producing materials					
State	Speed	Food	P1	P2	P3	P4	P5	P6	P1	P2	P3	P4	P5	P6
1	1.5	0.3	0.2	0	0	0	0	0	0.01	0	0	0	0	0
2	1.5	0.3	0.34	0.07	0	0	0	0	0.017	0.105	0	0	0	0
3	1.5	0.3	0.34	0	0	0	0	0	0.017	0	0	0	0	0
4	5	0.5	0.34	0.07	0	0	0	0	0.017	0.105	0	0	0	0
5	1.5	0.3	0.2	0	0	0	0.34	0	0.1	0	0	0	0.017	0
6	1.5	0.3	0.34	0.07	0	0	0.34	0	0.017	0.105	0	0	0.017	0
7	1.5	0.3	0.34	0	0	0	0.34	0	0.017	0	0	0	0.017	0
8	5	0.5	0.34	0.07	0	0	0.34	0	0.017	0.105	0	0	0.017	0

.
